# Supplementary material for: Combined Metabolome and Transcriptome Profiling Reveal Optimal Harvest Strategy Model Based on Different Production Purposes in Olive
Source: Foods. 2021 Feb 7;10(2):360. doi: 10.3390/foods10020360 (PMC7915097; doi:10.3390/foods10020360)
Supplement: Supplementary file 1 [file foods-10-00360-s001.zip › Supplementary Figures.docx]

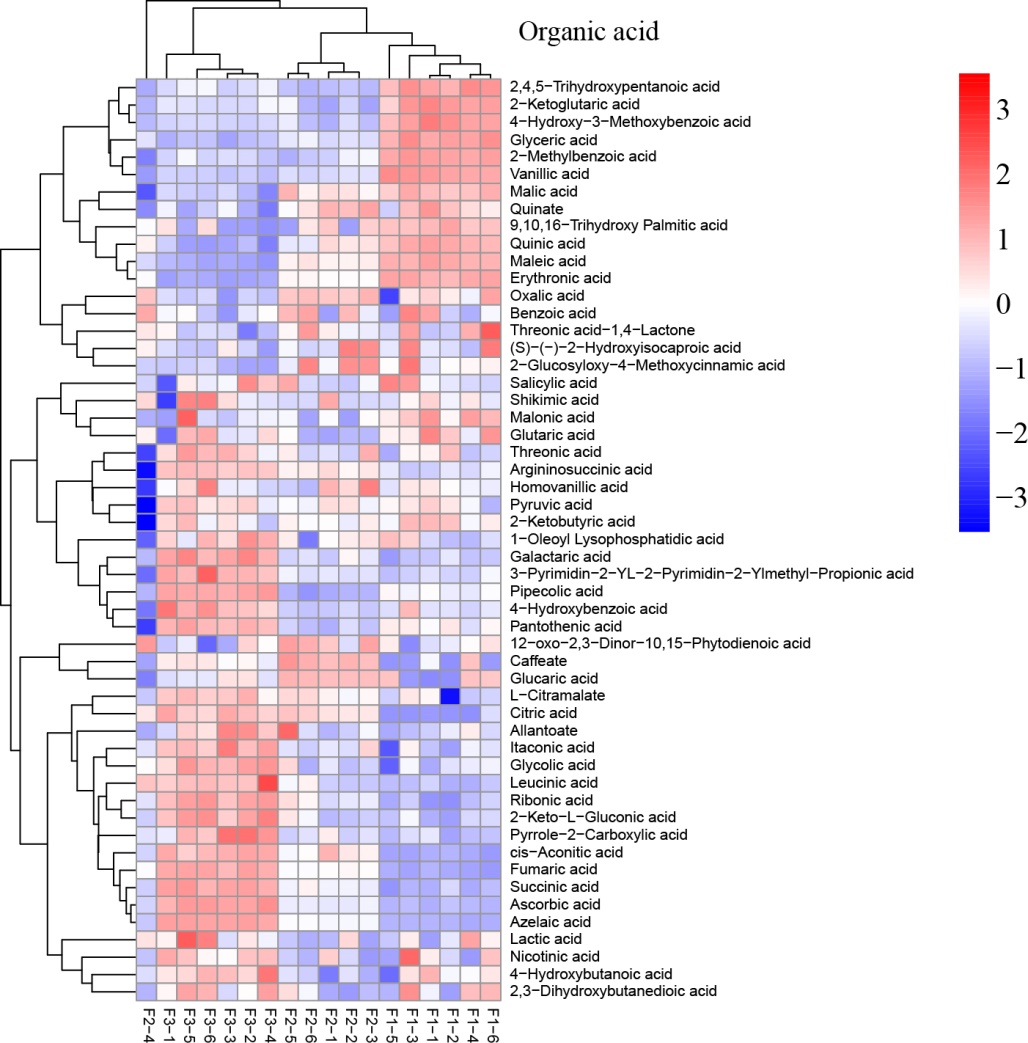


**Figure S1** Heat map visualization of organic acids among olive fruits at different maturity stages.


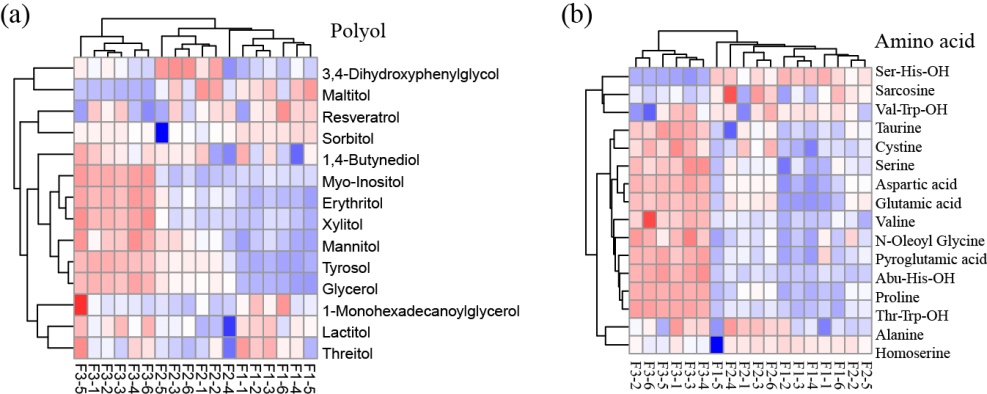


**Figure S2** Heat map visualization of amino (A) acids and (B) polyol among olive fruits at different maturity stages.


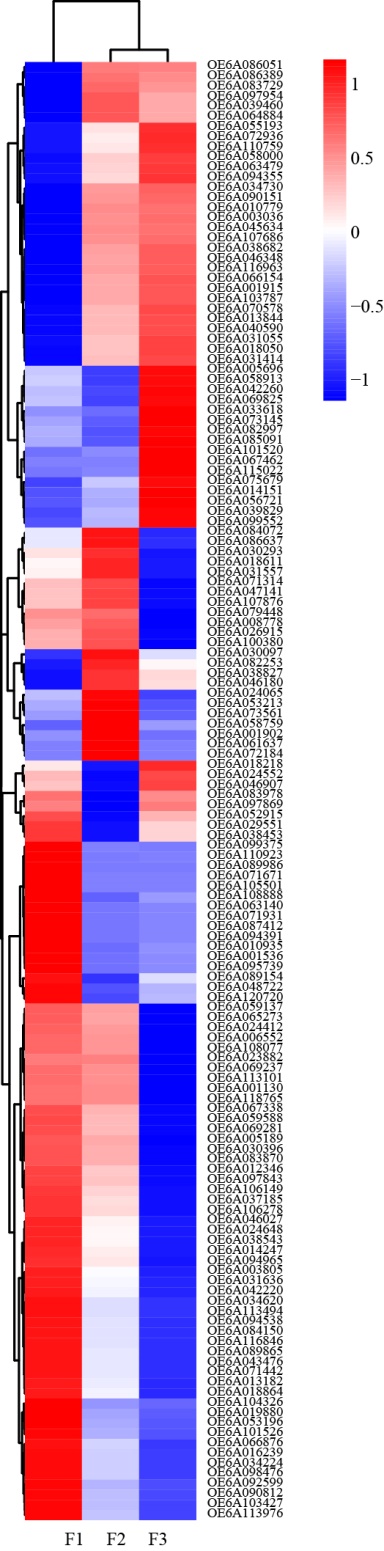


**Figure S3** Heat map visualization of ALDH family genes among olive fruits at different maturity stages.
